# Demographics, clinical characteristics, health resource utilization and cost of chronic thromboembolic pulmonary hypertension patients: retrospective results from six European countries

**DOI:** 10.1186/1472-6963-14-246

**Published:** 2014-06-09

**Authors:** Bernd Schweikert, David Pittrow, Carmine Dario Vizza, Joanna Pepke-Zaba, Marius M Hoeper, Anja Gabriel, Jenny Berg, Mirko Sikirica

**Affiliations:** 1 OptumInsight, Konrad-Zuse-Platz 11, D- 81829 Munich, Germany; 2 Institute for Clinical Pharmacology, Technical University, Dresden, Germany; 3 Department of Cardiovascular and Respiratory Science, School of Medicine, University of Rome “Sapienza”, Rome, Italy; 4 Papworth Hospital NHS Trust, Cambridge, United Kingdom; 5 Hanover Medical School, Hanover, Germany; 6 Bayer Pharma AG, Wuppertal, Germany; 7 OptumInsight, Stockholm, Sweden; 8 Bayer Pharma AG, Berlin, Germany

**Keywords:** Retrospective, Chart review, Pulmonary hypertension, Treatment, Cost

## Abstract

**Background:**

Chronic Thromboembolic Pulmonary Hypertension (CTEPH) results from incomplete resolution of a pulmonary embolus, leading to pulmonary hypertension and progressive right heart failure and death. We aimed to describe the demographics, treatment patterns, health resource utilization and related costs of patients with CTEPH.

**Methods:**

In specialized PH centres across six European countries, medical charts of CTEPH patients on PH medication were retrospectively extracted (chart review between 2006 and 2009). Resource utilization was valued using country-specific unit costs. Descriptive statistical analyses were performed.

**Results:**

Twenty-one hospitals documented 119 consecutive CTEPH patients over an average of 25.4 months. Patients were inoperable (83.9%) or persistent after surgery (16.0%) with mean age 67.5 ± 12.3 years, 61% were female. The average 6-minute walking distance was 298 ± 120 meters, and NYHA class II/III/IV was 27/59/14%. At baseline, 59.7% patients received endothelin receptor antagonist, 34.4% phosphodiesterase-5 inhibitors, and 5.8% prostacyclin. Adding a second PH medication was the most common regimen change. CTEPH patients experienced 1.8 ± 2.2 hospitalizations per year accounting for 14.8 ± 26.1 days in hospital. Patients paid on average 2.8 office visits per year to their general practitioner and 1.3 visits to a specialist. Unadjusted annual mortality rate was 6.0%. Annual cost of PH specific medication was the predominant economic factor averaging € 36,768 per year. Costs for hospitalizations (€ 4,496) and concomitant medications (€ 2,510) were substantially lower. Other health care resource items only accounted for marginal additional costs.

**Conclusion:**

CTEPH patients are characterised by substantial morbidity and mortality. Health care utilisation, predominantly due to off-label use of PH drugs, is significant.

## Background

Pulmonary hypertension (PH) is a debilitating disease of the pulmonary artery branches characterized by increased pulmonary arterial pressure and pulmonary vascular resistance [1,2]. The condition is often associated with progressive right ventricular failure and a poor prognosis. An important and cause of PH is chronic thromboembolic PH (CTEPH), which is the result of pulmonary vascular obstruction characterized by recurrent, unresolved pulmonary emboli and/or progressive pulmonary vascular thrombosis and scarring [3].

Prospective studies indicate that between 0.6% and 4.6% of acute pulmonary embolic survivors develop symptomatic CTEPH [4,5]. Furthermore, approximately 30% to 50% of CTEPH patients have been reported not having a history of acute venous thromboembolism [6,7].

Due to the rarity and complexity of the condition, patients with CTEPH according to international and national PH guidelines should be treated in expert centres only [1]. The treatment of choice for CTEPH is surgical pulmonary endarterectomy (PEA), which provides a potential cure of the disease, in particular if performed at expert centres with this surgical capability [8]. However, a substantial portion of patients may be considered ‘inoperable’ due to distal location of pulmonary thromboembolic or severe comorbidity and have a poor prognosis if untreated [9,10]. In addition, roughly 10% of patients who undergo PEA maintain a pulmonary hypertensive state since they obtain limited relief from surgery or experience recurrence [9]. Such inoperable or residual/recurrent patients are frequently treated with PH drugs (off-label) due to the lack of other treatment alternatives [11]. To date, positive randomized control trial evidence for medication use in CTEPH has only recently been demonstrated in the CHEST-1 study with riociguat [12]. Only very recently a drug has been approved for the treatment of CTEPH in Europe and the US. Additionally, there is a lack of data on costs and resource utilization associated with CTEPH in patients in the real-world setting.

Against this background, we aimed to describe the demographics, drug treatment patterns, outcomes and costs of patients with CTEPH treated under everyday practice conditions in six European countries.

## Methods

### Design and organisation

This present study was a retrospective chart review in 21 specialist centres in six countries: France (3 centres), Spain (4), Italy (4), UK (3), Sweden (1), and Germany (3). Data from consecutive patients were collected at each site for a maximum of up to 39 months after the initiation visit within the observation period between July 2006 and September 2009. The ethics review boards of each centre approved data collection, and data protection rules were closely observed (see Additional file 1 for a complete list of the involved ethic commissions and review boards). Patients were eligible for inclusion, if they were at least 18 years old, had a confirmed diagnosis of CTEPH (Group 4 according to Dana Point 2008 criteria), were in NYHA class II to IV, treated with monotherapy or combination therapy with endothelin receptor antagonists (ERA), prostacyclin analogues (PA) or phosphodiesterase-5 (PDE-5) inhibitors. The only exclusion criterion was HIV.

Written informed consent was obtained from the patients according to local regulations and in line with the recommendation of the responsible ethic committees and review boards.

### Patient variables

Information was collected on demographics (age, gender, employment status etc.), diagnostic information (type of CTEPH with differentiation between inoperable or operated patients with persisting and recurrent PH after PEA; time since first diagnosis), clinical data (New York Heart Association [NYHA] class, 6-minute walk distance, Borg dyspnoea index), hemodynamic and lung function (pulmonary artery pressure, pulmonary capillary wedge pressure, pulmonary vascular resistance, right atrial pressure, cardiac index, respiratory capacity), comorbidities and risk factors (smoking, alcohol consumption etc.). Detailed information was collected on treatments focussing on PH drugs, i.e. the endothelin receptor antagonists (ERA) ambrisentan, bosentan, and sitaxentan, the PDE-5 inhibitors sildenafil and tadalafil, or prostacyclines. Dosages were recorded. Further, co-medication with calcium channel blockers, diuretics and digoxin were recorded at the class level. With respect to outcomes, the following events were recorded: lung or heart lung transplantation, atrial septostomy, PEA, hospitalisation, ambulatory/outpatient centre visits, examinations and procedures, and other health care services.

### Data collection

Data were extracted from patient files by staff members of the individual sites according to guidelines provided for this procedure. Standardized case report forms were used. Data were entered into the database at a central site, and checked for plausibility and completeness. In case of missing data or queries, sites were contacted to resolve the issues. No on-site monitoring was performed.

### Statistical analysis

Due to the nature of the study and the small patient population, all statistical analyses were exploratory and used in a descriptive manner. Incidence rates, time to first event and standard deviations were provided for discrete variables. Means per year of follow-up and standard deviations were presented for continuous variables. For survival analysis, Kaplan-Meier estimates were used. Resource utilization was valued using country-specific unit costs.

## Results

### Disposition and characteristics

Of 119 CTEPH patients, 49 (41.2%) were documented in France, 34 (28.6%) in Germany, 17 (14.3%) in the UK, 9 (7.6%) in Italy, 7 (5.9%) in Spain, and 3 (2.5%) in Sweden. Mean observation time was 25.4 ± 25.6 months, and the mean number of recorded visits 7.1 ± 3.3. Baseline characteristics are summarized in Table 1. Patients were on average 67.5 ± 12.3 years old, women accounted for 60.5%. Time since PH diagnosis was 16.0 ± 47.3 months.

**Table 1:** Baseline characteristics

**Baseline variable**	Sample size (N)*	**Value**
Age, years (mean ± SD)	119	67.5 ± 12.3
Gender, female, %	119	60.5
BMI, kg/m^2^ (mean ± SD)	112	26.8 ± 5.4
Comorbidities, number	119	3.4 ± 1.9
NYHA class, (mean ± SD)	119	2.9 ± 0.6
class, II, III, IV (%)	119	27/ 59/ 14
6-min walk distance, (mean ± SD)	92	298 ± 120
History of PH, months (mean ± SD)	116	16.0 ± 47.3
Inoperable CTEPH, %	118	83.9

*Sample size may vary due to missing values in the patient records.

Comorbidities were prevalent (mean number per patient 3.4 ± 1.9), with the most frequent ones including arterial hypertension (41 patients, 34.2%), pulmonary embolism with or without acute cor pulmonale (30.0%), other pulmonary heart disease (17.5%), phlebitis and thrombophlebitis (14.2%), and heart failure or chronic obstructive pulmonary disease (10% each). Patients were defined as inoperable in 99 cases (83.9%) and as having persisting or recurrent PH after PEA in 19 cases (16.0%; 1 patient with missing information).

Mean 6-minute walking distance was low (298 ± 120 meters). Most patients were in NYHA classes II (26.8%) or III (58.8%), and fewer in IV (14.3%). Hemodynamic are displayed in Table 2. Mean pulmonary arterial pressure was 46 ± 11 mmHg, mean pulmonary vascular resistance 797 ± 416 dyn × sec × cm^−5^.

**Table 2:** Clinical outcomes and hemodynamics, baseline and change during follow-up

**Parameter**	**Baseline**		**Follow-up**	
	N*	**Mean ± SD**	N*	**Δ during observation (Mean ± SD)**
6 MWD, meters	92	298 ± 120	90	30 ± 90
Pulmonary atrial pressure (PAP),mmHg	107	45.5 ± 0.6	51	−2.5 ± 13.4
Pulmonary capillary wedge pressure, mm Hg	98	9.5 ± 4.6	43	−1.0 ± 5.9
Right atrial pressure (RAP), mmHg	96	8.1 ± 5.8	53	−0.43 ± 6.7
Pulmonary vascular resistance (PVR), dyn × sec × cm^−5^	94	797 ± 416	37	−111 ± 360
Cardiac index, l/min/m^2^	98	2.2 ± 0.5	49	1.11 ± 3.3
Borg dyspnoea index	81	4.7 ± 2.2	78	−0.5 ± 2.7
FEV_1_, litres	86	2.0 ± 0.7	71	−0.1 ± 0.3
FEV_1_/vital capacity	87	71.5 ± 12.3	72	−3.1 ± 10.4

*Sample size may vary due to missing values in the patient records.

FEV_1_, Forced Expiratory Volume in 1 second; 6-MWD, walking distance in 6 minutes.

### Medication

**Figure 1:**
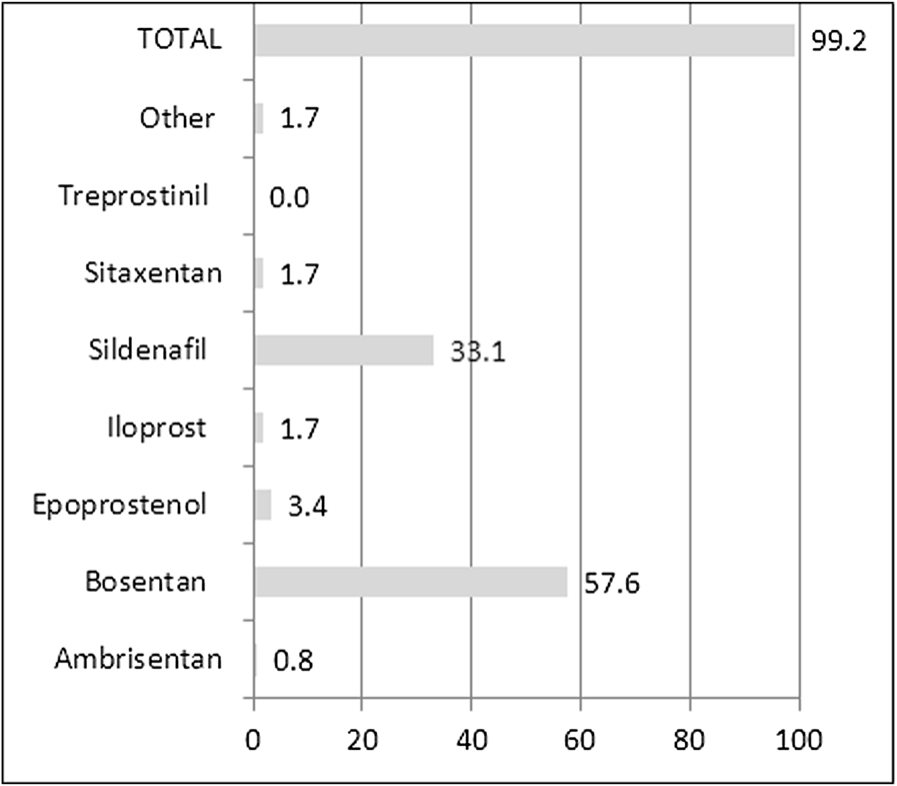
**PH-specific medication at inclusion (% of patients).** Medication for PH at time of inclusion (% of patients).

**Figure 2:**
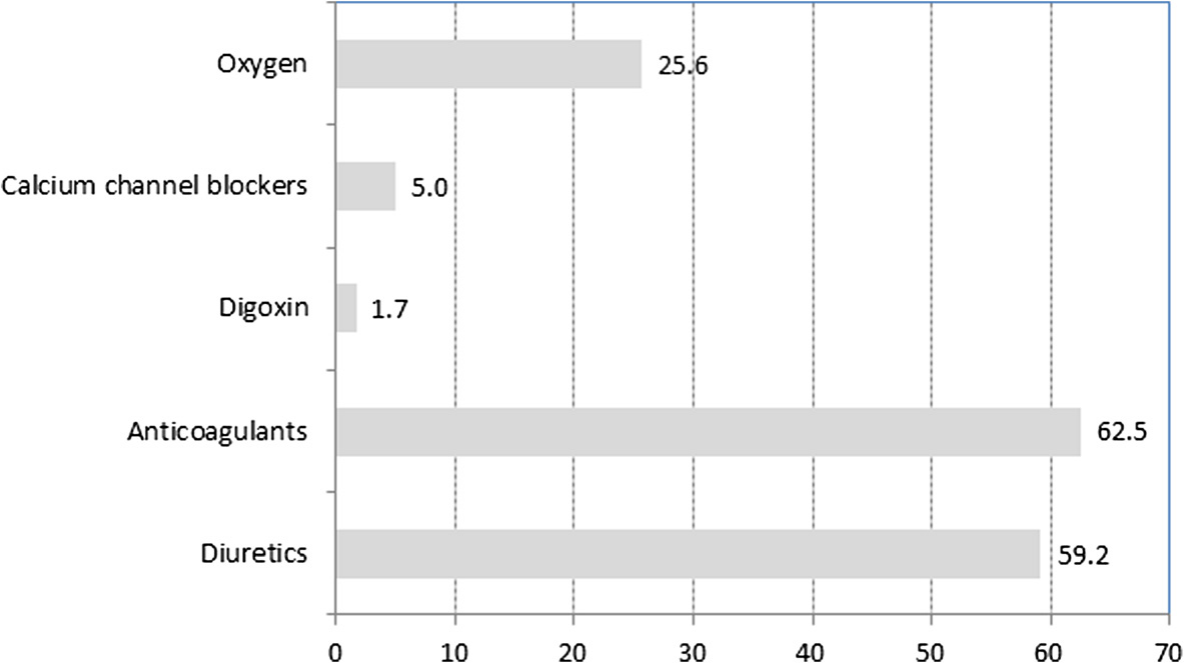
**Co-medication and accompanying treatment (% of patients).** Co-medication and accompanying treatment at time of inclusion.

**Figure 3:**
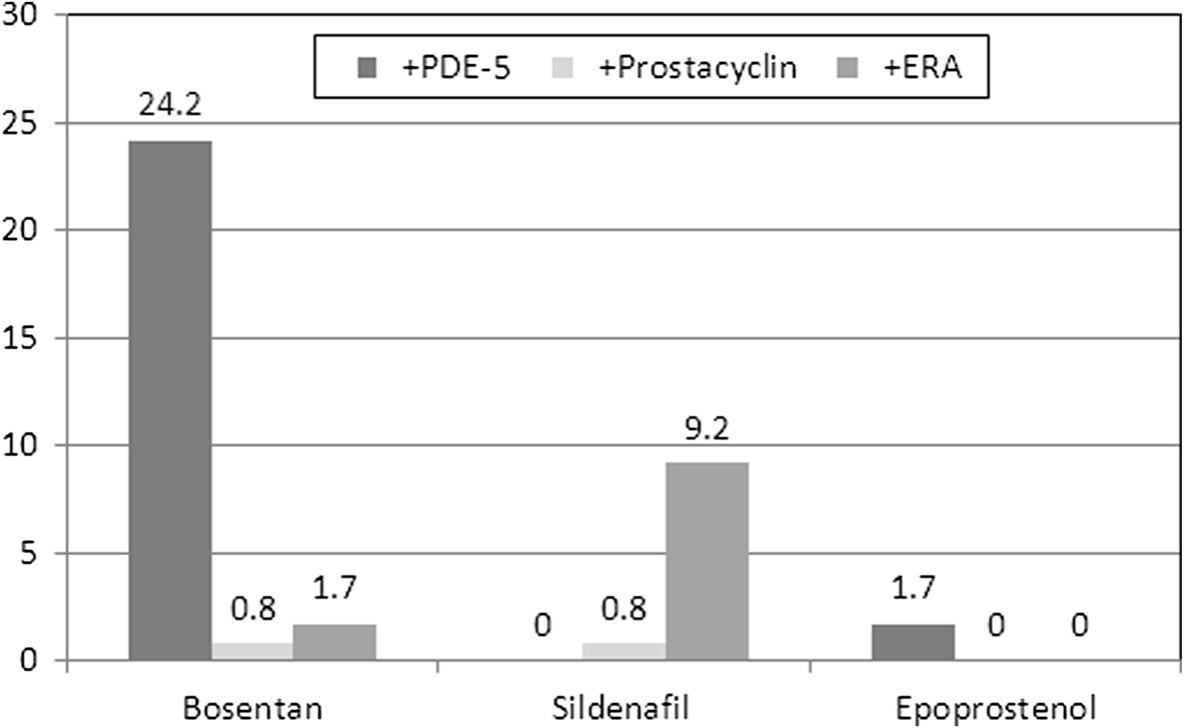
**Changes in treatment during follow-up (percent of patients).** Percentage of patients on medication for the three most common medications.

PH drugs were used off-label in CTEPH (Figure 1). At the time of the data collection, monotherapy prevailed, mostly with bosentan (68 patients, 57.6%), sildenafil (39 patients, 33.1%), or epoprostenol (3.4%). All other drugs were reported in less than 2% of patients. Combination therapy was reported in 1 patient only (ERA plus PDE-5 inhibitor). As concomitant medications, mainly anticoagulants (62.5%) and diuretics were noted (59.2%). Oxygen was used in every fourth patient (25.8%, Figure 2).Medication changes over time are shown in Figure 3. Discontinuation of the first therapy was infrequent (6 cases on bosentan and 6 cases on sildenafil, 5.0% each). Switch from bosentan to another ERA occurred in 16 cases (13.3%) and to a PDE-5 inhibitor in 5 cases (4.2%), while switch from sildenafil to ERA occurred only in 1 patient (0.8%), and to other PDE-5 inhibitors in 5 patients (4.2%). One patient on epoprostenol (0.8%) switched to another prostacyclin, and another patient to an ERA.

Adding a second PH medication was the most common regimen change. For bosentan, in 29 patients (24.2%) a PDE-5 inhibitor was added, in 2 cases (1.7%) another ERA, and in 1 case (0.8%) a prostacyclin. For sildenafil, in 11 cases (9.2%) an ERA was added and in 1 case (0.8%) a prostacyclin. For prostacyclin, in 1 case each (0.8% each) an ERA or a PDE-5 inhibitor was added.

### Clinical course

**Figure 4:**
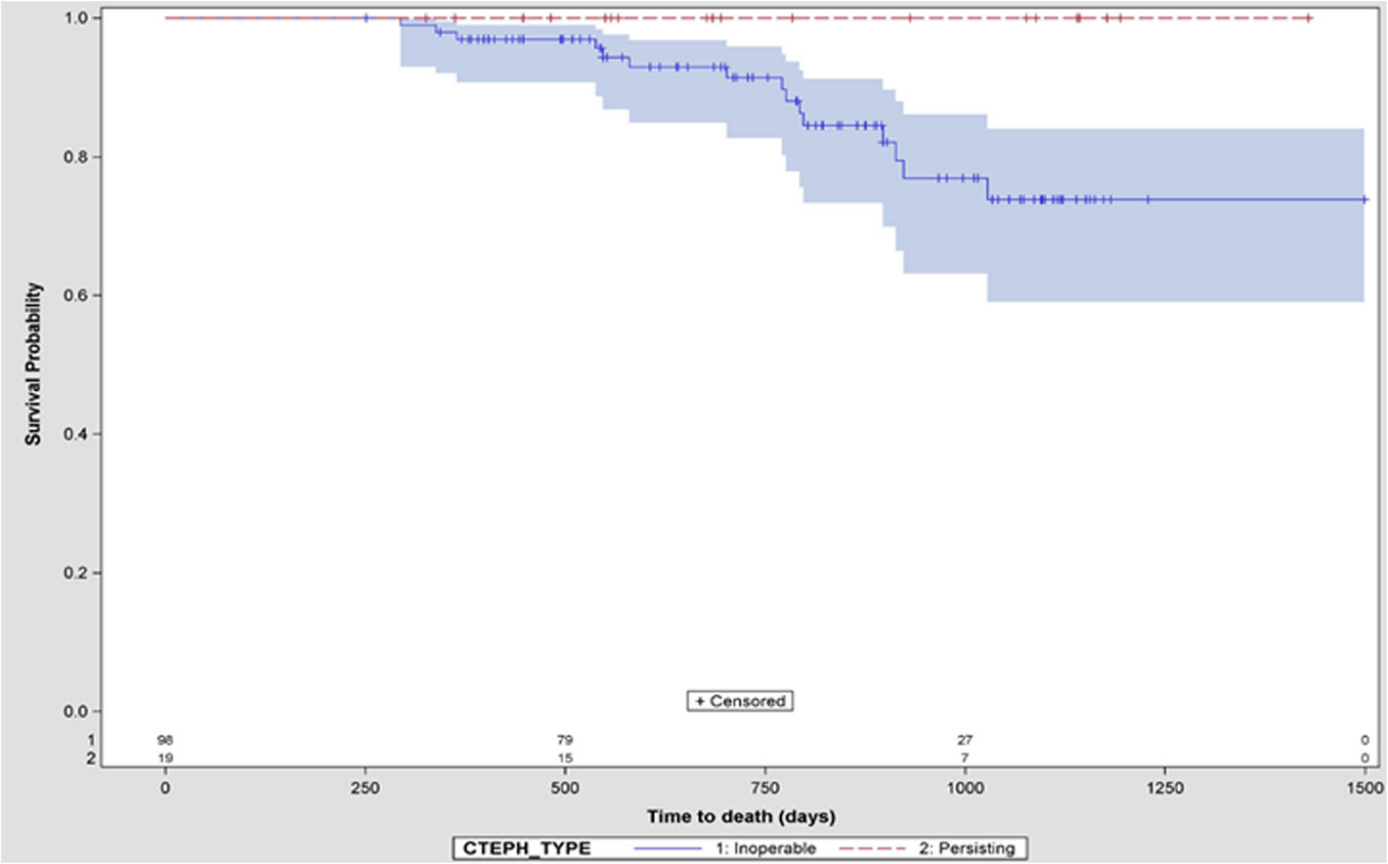
**Survival, by PEA status.** Kaplan-Meier curve of survival by status of pulmonary endartherectomy: blue = inoperable, red = persisting CTEPH after PEA.

Most patients were relatively stable during follow-up in terms of hemodynamics (Table 2) and events. Six-minute walking distance increased by 30.3 meters. At the last observation compared to baseline, 38 patients (31.9%) had improved with respect to NYHA class, 69% (58.0%) had remained stable, and 10 (8.4%) had deteriorated.Surgical interventions comprised 9 PEA (8.3%) and 1 atrial septostomy (0.8%). A total of 15 patients (12.6%) died during observation, with an unadjusted annual mortality rate of 6.0%. Time to death in inoperable CTEPH versus residual PH is shown in Figure 4, indicating a substantially higher mortality and significant unmet need for CTEPH patients who are not eligible for PEA surgery.

### Resource utilization and costs

**Figure 5:**
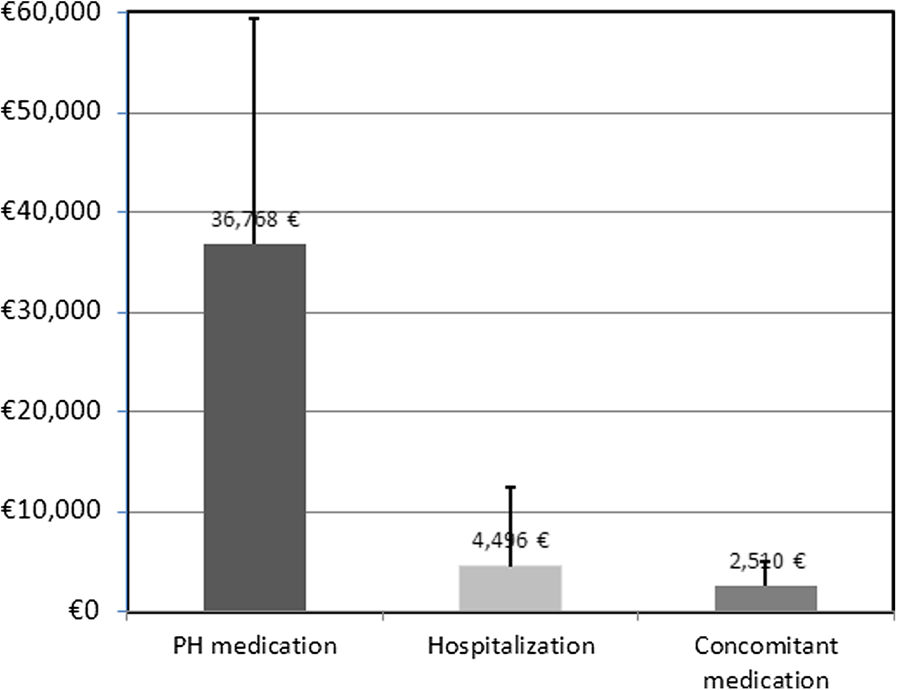
**Yearly costs related treatment of CTEPH.** Mean annualized cost of health care related to CTEPH treatment. Main treatment categories. Whiskers represent standard deviation.

Patients had on average 2.8 ± 4.2 office visits with their general practitioner and 1.3 ± 1.4 visits to a specialist per year (Table 3). Hospitalisations were frequent (1.8 ± 2.2 per patient and year), as were examinations or diagnostic tests (8.4 ± 5.9).Annual costs of PH medication was the predominant cost factor (36,768 € ± 22,630), followed by costs for hospitalisations (4,496 € ±7,923) and concomitant medications (2,510 € ±2,503, Figure 5). Costs for ambulatory visits to GPs and specialists were negligible (<100 € each per patient and year).

**Table 3:** Resource utilization

**Resource**	N*	**Mean ± SD**	**Median**
Hospitalizations per PY	118	1.8 ± 2.2	1.0
Hospitalization days per PY	74	14.8 ± 26.1	7.8
Time to first hospitalization (d)	74	146 ± 209	71.5
Examinations/tests per PY	118	8.4 ± 5.9	7.8
Visits to the GP per PY	64	2.8 ± 4.2	0.7
Visits to specialists per PY	77	1.3 ± 1.4	0.9
Medical aids per PY	48	0.6 ± 2.1	0

*Sample size may vary due to missing values in the patient records.

D = day; GP = general practitioner; PY = patient year.

Other health care resource items only accounted for marginal additional costs in both groups. Medical aids such as walking aids, inhalation devices or physiotherapy, were documented for one out of four patients.

## Discussion

The present analysis provides insight about the situation and resource utilisation of CTEPH patients in a sample of European specialist centres during the years 2006 to 2009. In line with a report of a large-scale European registry performed in 2007 to 2009 [6], our study shows that off-label treatment with PH drugs is common practice in patients with inoperable or residual CTEPH. Most patients were severely impaired as indicated by their NYHA class, exercise capacity (6-minute walk distance), hemodynamics and comorbidities. Compared to patients in the international prospective CTEPH registry performed in 2007–2009, patients in our cohort were somewhat older (68 versus 63 years), comprised more women (61% versus 50%), had a lower 6-minute walking distance (298 versus 329 meters), and a lower portion of inoperable patients (83% versus 36%) [6].

Drug treatment accounted for the largest share of direct costs for patients in the present chart review, while costs for hospitalisations and concomitant treatment were much lower, and costs for GP or specialists visits were negligible. To our knowledge, there are only two other studies that also reported the cost dimension of patients with CTEPH. First, Kirson et al. reported a series of 289 privately insured CTEPH patients in the US (mean age 52.2 years, 57.1% women), for whom mean direct costs per month (year 2007 values) were USD 4782 [USD 57,384 annualized] compared to USD 511 [6132/year] for controls, (p < 0.0001), and USD 2,023 [USD 24,276/year] for patients with pulmonary arterial hypertension (PAH) [13,14]. In that sample, inpatient services accounted for 54%, outpatient and other services for 33% and prescription drugs for 11% of total direct healthcare costs per patient-month in CTEPH patients [13]. Second, Said et al. estimated direct medical costs and resource use for commercially insured CTEPH patients during 2004–2009 within the US-American MarketScan database using a retrospective cohort design [15]. Compared to matched controls without CTEPH or PAH, CTEPH patients had significantly higher monthly costs and resource use (total costs 6198 USD [$74,376/year] versus 1,579 USD [$18,948/year], outpatient visits 1.2 versus 0.8, inpatient visits 2 versus 0.2, prescriptions 4.2 versus 2.8; all p-values <0.05) [15].

Other groups have published cost-utility [16], cost-effectiveness [17] or cost-minimisation analyses [18] for PAH (but not CTEPH) based on populations in the USA or Canada. For example, in a retrospective study similar to ours (claims database analysis) with 706 PAH patients in a large, geographically diverse US managed-care organization, PAH drugs were the main cost driver compared to PAH-related inpatient stays and emergency department visits, with average monthly costs of 5,332 USD for bosentan and 3,632 USD for sildenafil patients, respectively (p = 0.003) [19].

### Limitations of the study

A number of methodological considerations and limitations have to be taken into account when interpreting the current data. Data have to be interpreted carefully taking the principle limitations of a retrospective analysis into account, in particular: missing data, varying follow-up periods for patients, variation in reporting and documentation across sites and countries [20]. The comprehensive measurement of health care utilization is known to be challenging in studies which cannot rely on administrative data (preferably payer data).

It was anticipated that the availability of these types of utilization data depended on the country or even site-specific degree of service integration as well as the informational exchange across health care service providers (e.g. PH treatment centre and general practitioner). Missing data in particular with respect to medical aids were frequent. Therefore a total cost estimate could not be provided, as it would have been based on the small sample of patients when restricting aggregation to the patients with complete information. Relying on available information would have led to significant underestimation of total costs. However, the direct costs for medications outnumber other cost components by far.

Furthermore, the study focused on inoperable CTEPH patients treated with PH medication excluding patients that were not on PH medication. Although no PH medication had been approved for CTEPH at the time of the study, ERA, PA and PDE-5 inhibitors have already been widely used as suggested by guidelines as the treatment of choice. A further obvious limitation is the sample size of this study and the selection process using a centre-based approach. However, given the severity and rareness of the disease and the still limited number of observational studies investigating the condition, this study adds to the knowledge base by reporting clinical and economic outcomes from a real world sample.

## Conclusions

While CTEPH is the only form of PH that currently can be potentially cured, a substantial portion of patients are either inoperable or decline operation, or experience recurrent PH after the PEA. Physicians face a dilemma when treating such patients, as no drug has consistently demonstrated efficacy and received regulatory approval for the treatment of CTEPH. In the present study, CTEPH patients who were inoperable had a significantly worse prognosis compared to patients post-PEA. CTEPH patients had high resource utilization and costs, with off-label PH medications accounting for the highest costs. More research is required in the real-world setting to understand the cost implications of using off-label medications without demonstrated efficacy in randomized controlled trials.

## Abbreviations

CTEPH: Chronic thromboembolic pulmonary hypertension; ERA: Endothelin receptor antagonist; EUR: Euro; NYHA: New York Heart Association; PA: Prostacyclin analogues; PEA: Pulmonary endatherectomy; PDE-5: Phosphodiesterase-5; PAH: Pulmonary arterial hypertension; PH: Pulmonary hypertension; UK: United Kingdom; USD: US dollars.

## Competing interest

The study was sponsored by Bayer Pharma AG. BS and JB are employees of OptumInsight who were paid consultants to Bayer Pharma AG in connection with the development, conduct and disseminations of this study. DP received grants from Bayer Pharma AG for consultation services. DCV has received financial grants from Bayer Pharma AG, Actelion, GSK, Pfizer, Italfarmaco, Dompè, UTEL, and Lilly for lectures and consultation services. MH has received financial grants from Bayer Pharma AG, Actelion, GSK, Lilly, Novartis and Pfizer for consultation services. JPZ has received reimbursements of travel expenses to congresses and speakers' fees from Actelion, Pfizer, GSK, Bayer Pharma AG, Lilly and United Therapeutics, has participated to advisory boards for Actelion, Bayer Pharma AG, GSK, Pfizer, Lily, and has received funds for research/education from Actelion, Pfizer, Bayer Pharma AG, GSK. AG and MS are employees of Bayer Pharma AG.

## Authors’ contributions

BS participated in the design of the study, co-managed data collection and study conduct and carried out the statistical analysis. DP participated in design and analysis of the study and drafted the manuscript. DCV, JPZ and MH provided medical expertise, participated in the coordination of data collection and study conduct and interpretation of results. AG conceived the study and participated in its design coordination. JB participated in the design and coordination of the study and the statistical analysis. MS participated in study coordination, and analysis. All authors read and approved the final manuscript.

## Pre-publication history

The pre-publication history for this paper can be accessed here:

http://www.biomedcentral.com/1472-6963/14/246/prepub

## Supplementary Material

Additional file 1 List of involved ethic committees and review boards by country. Click here for file
