# Supplementary material for: Demographics, clinical characteristics, health resource utilization and cost of chronic thromboembolic pulmonary hypertension patients: retrospective results from six European countries
Source: BMC Health Serv Res. 2014 Jun 9;14:246. doi: 10.1186/1472-6963-14-246 (PMC4069093; doi:10.1186/1472-6963-14-246)
Supplement: Additional file 1 — List of involved ethic committees and review boards by country. [file 1472-6963-14-246-S1.pdf]

Demographics, clinical characteristics, health resource utilization and cost of chronic thromboembolic pulmonary hypertension patients: retrospective results from six European countries

List of involved ethic committees and review boards by country

EC/ Institution	Action
France	
CNIL (Commission Nationale de l'Informatique et des Libertés, Paris	approved data privacy
CNOM (Conseil National de l'Ordre des Médecins), Paris	approved contracts and fees
CCTIRS (Comité consultatif sur le traitement de l'information en matière de recherche dans le domaine de la santé)	approved justification of the data collection
Spain	
CEIC Galicia	approved
CEIC Hospital Universitario La Fe	approved
CEIC Hospital Universitario San Cecilio	approved
CEIC Illes Balears	approved
Italy	
Comitato Etico Indipendente Azienda Ospedaliero-Universitaria Degli Ospedali Riuniti Di Trieste	approved
Comitato Etico dell'Azienda Ospedaliera Vincenzo Monaldi, Napoli	approved
Comitato di Bioetica dell'Azienda Ospedaliera Ospedali Riuniti di Bergamo	approved
Comitato Etico dell'Azienda Policlinico Umberto I, Roma	approved
United Kingdom	
NHS Greater Glasgow and Clyde, West of Scotland Research Ethics Committee 4	Decision: approval not required under NHS arrangements
Sweden	
Regionala etikprövningsnämnden i Göteborg	approved
Germany	
Ethikkommission der Universität Greifswald	approved
Ethikkommission der Universität Dresden	notified
Ethikkommission der Medizinische Hochschule Hannover	notified
